# Bewegungsanalyse bei Querschnittverletzungen: Hilfestellung in der klinischen Entscheidungsfindung

**DOI:** 10.1007/s00132-023-04409-y

**Published:** 2023-07-25

**Authors:** Inga Kröger, Hannes Wackerle, Doris Maier, Orpheus Mach, Peter Augat

**Affiliations:** 1 Institut für Biomechanik, Berufsgenossenschaftliche Unfallklinik Murnau, Professor-Küntscher-Straße 8, 82418 Murnau, Deutschland; 2 Institut für Biomechanik, Paracelsus Medizinische Privatuniversität Salzburg, Salzburg, Österreich; 3 Zentrum für Rückenmarkverletzte, Berufsgenossenschaftliche Unfallklinik Murnau, Murnau, Deutschland

**Keywords:** Biomechanik, Ganganalyse, Gangstörung, neurologische, Paraplegie, Tetraplegie, Biomechanics, Gait analysis, Gait disorder, neurologic, Paraplegia, Tetraplegia

## Abstract

Für motorisch inkomplette Querschnittgelähmte ist eine Verbesserung der Gehfunktion ein wichtiges Ziel im Rehabilitationsprogramm. In den spezialisierten Behandlungszentren dienen der 6‑Minuten-Gehtest, der 10-Meter-Gehtest oder der „Timed-up-and-go“-Test als standardisiertes Messinstrument zur Erfassung der Gehfähigkeit. Diese Tests sind jedoch nicht in der Lage, die Qualität des Gehens zu beurteilen. Mithilfe der markerbasierten Bewegungsanalyse kann das Gangbild reliabel erfasst und die Qualität der Bewegung beurteilt werden. Dies ermöglicht eine objektive Bewertung der Gangqualität über den Verlauf der Jahre oder kann unterstützend für die Therapieplanung eingesetzt werden. Am Beispiel von zwei Fällen wird der Nutzen einer solchen Analyse vorgestellt.

## Abstract (en)

For motor incomplete spinal cord injured patients, improvement of walking function is an important aim in the rehabilitation program. In specialized treatment centers, the 6‑minute walking test, the 10-meter walking test or the timed-up-and-go test are used as an assessment tool to determine walking ability, but these tests are not able to assess the quality of gait. Marker-based movement analysis can be used as a reliable method to evaluate the gait pattern. This allows an objective assessment of gait quality over time or can be used to support therapy planning. The benefit of such an analysis is presented by means of two case studies.

Ein Teil der Menschen mit inkompletter Querschnittlähmung hat die Möglichkeit, die Fähigkeit zu Gehen wieder zu erlangen. Um die Qualität des Gangbilds zu beurteilen und den Erfolg durch den Einsatz verschiedener Therapieformen oder Hilfsmittel zu evaluieren, sind objektive Messverfahren, wie beispielsweise die instrumentelle Bewegungsanalyse, notwendig. Während im Rahmen von Forschungsprojekten die instrumentelle Bewegungsanalyse bei Querschnittgelähmten schon breite Anwendung findet, wird sie im klinischen Alltag hingegen nur wenig eingesetzt.

## Hintergrund Querschnittlähmung

Die weltweite Inzidenz von Querschnittlähmungen wird auf 40–80 Fälle/1.000.000 pro Jahr geschätzt [29]. In Deutschland wird von 11 Fällen/1.000.000 pro Jahr ausgegangen [15]. Von 2012–2021 wurden beispielsweise in der Berufsgenossenschaftlichen (BG) Unfallklinik Murnau, einem spezialisierten Zentrum für die Behandlung von Patienten mit Verletzungen des Rückenmarks, knapp 1400 Patienten mit einer akuten Querschnittlähmung behandelt, 76 % der Betroffenen waren Männer. Die Hauptursachen für die Rückenmarkaffektionen waren zu drei Vierteln traumatische Ereignisse, deutlich seltener fanden sich in unserem Klientel nichttraumatische Ursachen (z. B. Entzündungen, Ischämien, Hämorrhagien, Bandscheibenvorfälle).

Bei Querschnittlähmungen kann zwischen motorisch kompletten und inkompletten Lähmungen unterscheiden werden. Drei Monate nach Lähmungseintritt wurde bei über der Hälfte der sich in stationärer Behandlung befindenden Patienten nach dem ISNCSCI-Protokoll (International Standards for Neurological Classification of Spinal Cord Injury) eine motorisch inkomplette Lähmung AIS-Grad C‑D (American Spinal Injury Association Impairment Scale) diagnostiziert. Von diesen Patienten war ein Viertel gehfähig, wenn auch zum Großteil mit Unterstützung durch Hilfsmittel. Bei Personen mit inkompletter Querschnittlähmung sind die Aktivitäten des täglichen Lebens, wie beispielsweise das Gehen, durch Schädigungen der afferenten und efferenten Nervenfasern beeinträchtigt [30]. Schädigungen der efferenten Nervenfasern können sich durch verminderte Muskelkraft oder hyperaktiven Wirbelsäulenreflexen zeigen, Schädigungen der afferenten Nervenfasern führen zu einer beeinträchtigten Propriozeption und Sensorik [4, 30, 31]. Beide Systeme zusammen werden für eine ungestörte posturale Kontrolle benötigt [13].

Ebenso können Einschränkungen in der Gelenkbeweglichkeit durch Kontrakturen der Skelettmuskulatur entstehen [7]. Es können beispielsweise eine Missinterpretation der Oberflächensensibilität im Bereich der Fußsohle, fehlende Information zur Gelenkstellung (Tiefensensibilität), oder eine verminderte Gelenkbeweglichkeit aufgrund eingeschränkter Muskelkraft oder Kontraktur entstehen, die Abweichungen im Gangbild wie Stolpern oder Zehenschleifen hervorrufen [23]. Studien, in denen spezifische Gangcharakteristika z. B. hinsichtlich der Gelenkkinematik bei inkomplett Querschnittgelähmten beschrieben werden, gibt es kaum. Gil-Agudo et al. beschreiben eine größere Knieflexion zum Zeitpunkt des initialen Bodenkontakts sowie eine deutliche Verringerung der Plantarflexion im „toe-off“ bei Patienten mit Central-Cord-Syndrom [10]. Dahingegen wurden Veränderungen räumlich-zeitlicher Parameter wie eine verminderte Geschwindigkeit oder eine reduzierte Schrittlänge schon öfter berichtet [10, 20]. Die Angst zu stürzen ist bei Personen mit Querschnittverletzung im Vergleich zu gesunden Personen deutlich erhöht, auch das tatsächliche Auftreten von Stürzen ist erhöht [30, 31].

Die soziale Teilhabe ist als eines der Endziele eines umfassenden Rehabilitationsprozesses anerkannt [22]. Wenig überraschend, tauchen für eine höhere Teilhabe am sozialen Leben immer wieder Begriffe im Umfeld der Aktivität und Reduktion der körperlichen Behinderung auf, aber auch Eigenständigkeit und psychische Faktoren spielen hier eine Rolle [28]. In all diesen Punkten spielt das Wiedererlangen, beziehungsweise die Verbesserung von Gehfunktionen eine wesentliche Rolle [1, 21, 26]. Die Gehfähigkeit wird bei inkomplett Querschnittgelähmten anhand der WISCI-Skala (Walking Index for Spinal Cord Injury) beurteilt [9]. Sie dient jedoch nicht der Beurteilung von Qualität und Funktionalität des Gangbilds, sondern erfasst lediglich die hierfür benötigte Unterstützung in Form von Hilfsmitteln oder Hilfspersonen [8]. Auch die üblicherweise in spezialisierten Zentren durchgeführten Gangtests wie der 6‑Minuten-Gehtest geben nur Auskunft über quantitative Werte, dienen jedoch nicht der qualitativen Beurteilung [12]. Hier kann die markerbasierte Bewegungsanalyse ansetzen und eine wichtige Grundlage zur Entscheidungsfindung für eine Hilfsmittelversorgung sowie eine Therapiezielsetzung darstellen.

In der BG Unfallklinik Murnau wurden seit 2016 insgesamt 250 inkomplette Tetra- und Paraplegiker zur Unterstützung des Therapiemanagements mithilfe der instrumentierten Bewegungsanalyse evaluiert. Insbesondere nach Abschluss der Erstrehabilitation und Plateaubildung der neurologischen Regeneration sollten sich die Betroffenen einer objektiven Untersuchung unterziehen können. Viele Patienten werden zu diesem Zeitpunkt jedoch nicht mehr ausführlich nachuntersucht [24]. Die nach wie vor unzureichende bis fehlende Kostenübernahme ist hier einer der Hauptgründe [18].

## Bewegungsanalyse als Hilfe für die klinische Entscheidungsfindung

Gangbilder von Tetra- und Paraplegikern lassen sich nicht per se klassifizieren, die Bandbreite ist groß und somit wird eine individuelle Begutachtung notwendig [11]. Eine Klassifizierung der einzelnen Lähmungshöhen, basierend auf kinematischen oder kinetischen Parametern, war somit in der Literatur nicht zu finden. Um die Wirkung von Maßnahmen wie aktiven oder passiven Orthesen, Physiotherapie oder Botoxinjektionen objektivieren zu können, ist die instrumentelle Bewegungsanalyse als reliables Messinstrument geeignet. Auch zur Evaluation in der Entwicklung von Hilfsmitteln wird die instrumentelle Bewegungsanalyse eingesetzt [2, 3, 16, 20]. In der klinischen Entscheidungsfindung kann sie ebenso einen wertvollen Beitrag leisten.

Die dreidimensionale Bewegungsanalyse kann objektive und quantifizierbare Informationen liefern

Nur wenige Bewegungsanalyselabore haben sich jedoch auf die Untersuchung von Erwachsenen mit neurologischen Erkrankungen spezialisiert [18]. Systematische Untersuchungen zum klinischen Nutzen der Bewegungsanalyse bei inkompletten Querschnittlähmungen gibt es bisher wenig, nichtsdestotrotz zeigen einige Arbeiten einen deutlichen Zugewinn für die Behandlungsplanung und somit letztendlich auch für die Betroffenen selbst. In diesem Zusammenhang lassen sich mithilfe der markerbasierten Analyse und korrelierenden Werten aus der körperlichen Untersuchung primäre Ganganomalien von sekundären kompensatorischen Mechanismen differenzieren [18, 20]. Ebenso werden leichte Einschränkungen, die mittels Funktionsuntersuchung festgestellt werden, unter Belastung verdeutlicht, wodurch z. B. eine fehlende dynamische Kontrolle sichtbar wird [24].

Die dreidimensionale Bewegungsanalyse kann spezifische Informationen liefern, um gezielte Managementpläne zur Optimierung des Gangs von Menschen mit inkompletten Querschnittverletzungen zu erstellen. So kann sie helfen, zwischen einer Gangveränderung aufgrund von Spastik, Muskelschwäche oder Kontrakturen zu unterscheiden und somit beispielsweise die Entscheidung zwischen einer Orthesenversorgung oder einem Spastikmanagement erleichtern [18, 20].

Die im klinischen Alltag verwendeten Skalen zur Bewertung einer Spastik (Ashworth oder Tardieu) sind nicht sensitiv genug, um die Wirkung von Botulinumtoxin auf das Gangbild hinreichend zu beurteilen [5]. Mittels einer Bewegungsanalyse vor und nach einer solchen Injektion kann die Wirkung auf das Gangbild objektiv überprüft werden. Die Studie von Bernuz et al. zeigte, dass bei inkomplett Querschnittgelähmten AIS D mit einer Spastik des M. rectus femoris die Knieflexion in der Schwungphase nach einer Injektion von Botulinumtoxin um 4° gesteigert werden konnte. Zusätzlich erhöhte sich die Knieflexionsgeschwindigkeit zum Zeitpunkt des „toe-offs“ um 13 % [5].

Murphy et al. [20] haben den Beitrag der Bewegungsanalyse zur klinischen Entscheidungsfindung untersucht. Die häufigsten Gründe für die Durchführung einer Bewegungsanalyse waren die Identifizierung von Therapieoptionen (35 %), gefolgt von der Ermittlung der Ursache von Gangstörungen und Optimierung der Behandlung (19 %), die Auswahl der Muskeln für eine Botulinumtoxin-A-Therapie (15 %) und die Auswirkungen der Spastik und/oder Muskelschwäche auf den Gang (13 %). Resultierend aus den Gangmessungen wurde am häufigsten (bei 22 von 48 der Betroffenen) eine Versorgung mit einer AFO (engl. „ankle-foot orthosis“) zur Stabilisierung der Standphase und/oder Gewährleistung der Bodenfreiheit in der Schwungphase empfohlen. Jeweils 8 von 48 Patienten wurde die Therapie mit Botulinumtoxin A oder eine systemische Spastikbehandlung empfohlen. Bei knapp 15 % der Patienten wurde die ursprünglich geplante Therapie aufgrund der Erkenntnisse aus der Bewegungsanalyse gänzlich verändert [20].

### Methodik der Bewegungsanalyse in der BG Unfallklinik Murnau

Die instrumentierte Bewegungsanalyse wird an der BG Unfallklinik Murnau mit acht Infrarotkameras (MX T20s, Vicon, Oxford, UK) bei einer Messfrequenz von 200 Hz durchgeführt. Mithilfe eines biomechanischen Modells (Plug-in-Gait-Markerset) werden Bewegungen des Rumpfes, des Beckens sowie der unteren Extremitäten gemessen. Die Erfassung der Kinetik erfolgt mit zwei synchronisierten, in den Boden eingelassenen Kraftmessplatten (OR6-7-2000, AMTI, Watertown, MA, USA) mit einer Messfrequenz von 1000 Hz. Zusätzlich wird eine synchronisierte zweidimensionale Videoanalyse durchgeführt. Zur Auswertung wird ein Mittelwert aus fünf Gangzyklen pro Seite gebildet.

Inkomplett Querschnittgelähmte mit einer AIS-C- oder -D-Klassifikation werden mittels 3‑D-Bewegungsanalyse an der BG Unfallklinik Murnau untersucht. Eine Stehfähigkeit von 15 min sowie eine Gangstrecke von 100 m müssen durchführbar sein. Die Patienten dürfen maximal auf Unterarmstützen angewiesen sein, ein Gehen am Rollator gilt als Ausschlusskriterium für eine markerbasierte Bewegungsanalyse. Die Anmeldung zur Bewegungsanalyse für inkomplett Querschnittgelähmte geht mit einer Fragestellung der behandelnden Ärzte, der Physiotherapeuten oder Orthopädietechniker einher. Die Daten werden von erfahren Bewegungswissenschaftlern aufbereitet und vorab interpretiert. Gemeinsam mit dem behandelnden Team aus Therapeuten, Ärzten und bei Bedarf Orthopädietechnikern werden die endgültigen Handlungsempfehlungen abgesprochen und mit den Zielen und Möglichkeiten des Patienten abgestimmt.

Jede Bewegungsanalyse wird durch eine Anamnese sowie eine Funktionsuntersuchung ergänzt. Hierbei wird der passive Bewegungsumfang der unteren Extremität mittels Goniometer erfasst. Muskelfunktionswerte für die untere Extremität werden anhand des 6‑stufigen Tests nach Janda durchgeführt [27]. Anhand der Ashworth-Skala wird die Spastizität bewertet [32]. Die Überprüfung der Bewegungsempfindung der Patienten erfolgt mit geschlossenen Augen [17]. Für die abschließende Interpretation werden die Daten der Anamnese, des Funktionsbefundes sowie die der instrumentellen Bewegungsanalyse in Zusammenhang gebracht. Anhand der nachfolgenden Fallbeispiele soll dieses Vorgehen dargestellt werden.

### Fallbeispiel 1

Im Rahmen einer stationären Rehabilitation stellte sich 2022 der 56-jährige Patient im Ganglabor vor. Die klinische Fragestellung war eine Statuserhebung des Gangbildes mit der Bitte um Therapieempfehlung. Es besteht die Diagnose einer inkompletten Tetraplegie sub C4 AIS C bei Zustand nach Polytrauma von 1992. Zudem wurde 2011 nach posttraumatischer Gonarthrose rechts eine Knie-Totalendoprothese implantiert. Berichtet wird über die Gangbildveränderungen zwischen der Messung ohne Orthese und der Messung nach 2‑wöchiger Erprobung einer Sprunggelenksorthese.

Bei der Vorstellung im Bewegungsanalyselabor hat sich nach Angaben des Patienten die maximale Gehzeit unter Verwendung von Unterarmgehstützen in letzter Zeit von circa 30 min auf 10 min reduziert. Insgesamt sei das Kraftniveau gegenüber dem letzten Aufenthalt in der Klinik verringert.

Im Muskelfunktionsbefund nach Janda [27] zeigte das rechte Bein bessere Kraftwerte als die Gegenseite (Tab. 1). Die passive Beweglichkeit der Gelenke der unteren Extremität war weitestgehend unauffällig (leichte Einschränkung Knieflexion links und Hüftaußenrotation und -flexion rechts), die Tiefensensibilität war linksseitig normal und rechts eingeschränkt. Spastiken zeigte der Patient in den linken Plantarflexoren (Ashworth 3). Der Patient war zum Zeitpunkt der Messungen mit Unterarmgehstützen und einem Rollstuhl für längere Gehstrecken versorgt. Die Gang- und Stehfähigkeit ist nur mithilfe von Unterarmgehstützen möglich.

**Table 1:** 

	Links	Rechts
M. iliopsoas	1	4
M. gluteus maximus	1	4
M. gluteus medius	1	4
M. quadriceps femoris	1	5
Mm. ischiocrurales	1	3
M. gastrocnemius	1	2
M. tibialis anterior	2	3
M. peroneus	1	3

**Figure 1:**
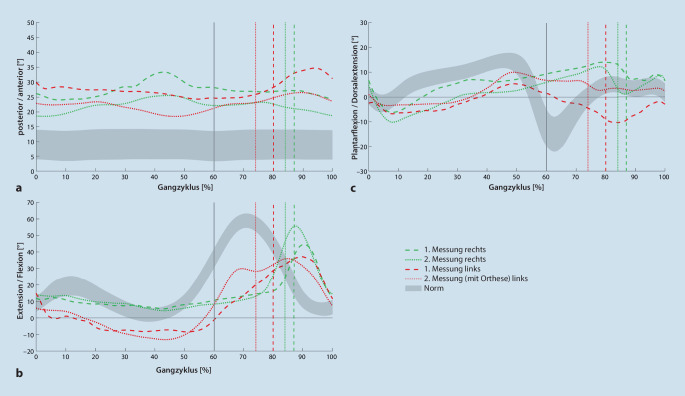

Als Hauptproblem wurde in der Bewegungsanalyse eine eingeschränkte Kontrolle des linken Sprung- und Kniegelenks identifiziert. Der Fußaufsatz des linken Beines erfolgte als Beistellschritt im „foot flat“. Dies wurde der zu schwachen prätibialen Muskulatur sowie der starken Spastik der Plantarflexoren, die einen physiologischen Fußaufsatz über die Ferse verhindert, zugeordnet. Im weiteren Verlauf der linken Standphase (ca. 10–60 % des Gangzyklus) befindet sich das linke Knie in Hyperextension (Abb. 1). Als primäre Ursache wird hierfür die Spastik der Plantarflexoren angesehen: Durch den erhöhten Tonus ist die Abrollbewegung des Sprunggelenks nicht möglich, neben der Plantarflexion wird das Kniegelenk in Hyperextension gezogen [25]. Weitere mögliche Gründe für eine Hyperextension können in einer Kombination aus zu geringer Kraft des M. quadriceps femoris und der Knieflexoren gesehen werden.

Die Knieextensoren sollten eine Beschleunigung in Richtung einer Flexion verringern und im „mid stance“ eine Überstreckung verhindern. Die verminderte Aktivität der Knieflexoren (Hamstrings und M. gastrocnemius) führt zu einem verringerten Flexionsmoment, eine Hyperextension im Kniegelenk kann nicht verhindert werden [6, 19]. Das Kniegelenk wird nur passiv über die Bandstrukturen gehalten. Der Patient steht während des gesamten Ganges in deutlicher anteriorer Beckenkippung. Die Einleitung der Schwungphase geschieht aufgrund eines zu schwachen M. iliopsoas über eine nach dorsal gerichtete Beckenkippung. Die Bodenfreiheit ist in der Schwungphase aufgrund unzureichender Kraft und der resultierenden verminderten Knie- und Dorsalflexion stark reduziert (Abb. 1). Um ein Mindestmaß an Bodenfreiheit zu erlangen, befindet sich das linke Becken in Elevation bei gleichzeitiger Lateralflexion des Rumpfes nach links. Trotz der Ausweichbewegungen war ein Zehenschleifen des linken Fußes bei zeitgleicher Supinationsstellung in der ipsilateralen Schwungphase auffällig, wodurch die Gefahr des Hängenbleibens des linken Fußes am rechten Standbein bestand. Der Patient berichtete daher von einer notwendigen erhöhten Aufmerksamkeit und Konzentration, um Stürze zu verhindern. Trotz der geringen Ganggeschwindigkeit und der Konzentration stürzte der Patient in jüngster Zeit vermehrt.

**Figure 2:**
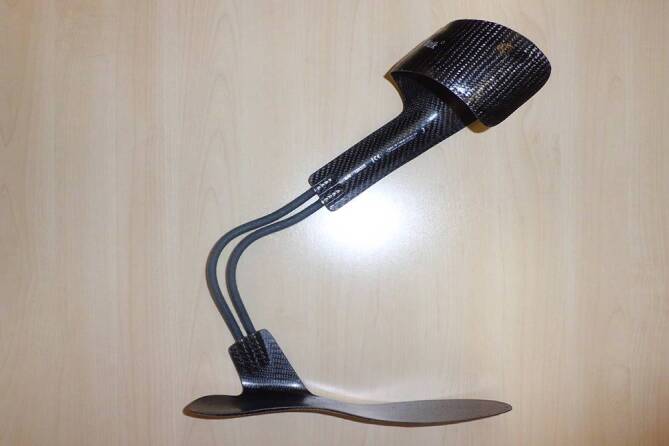

Aufgrund der Ergebnisse der Bewegungsanalyse wurde im interdisziplinären Team entschieden, den Patienten mit einer Sprunggelenkorthese links zu versorgen. Aufgrund des insgesamt schlechten Muskelstatus der linken Seite wurde zunächst von einer Spastikbehandlung abgesehen, es wurden im Rahmen der Therapie u. a. physikalische und physiotherapeutische Maßnahmen auf neurophysiologischer Grundlage und funktionelle Elektrostimulationen zur verbesserten Ansteuerung der Muskulatur durchgeführt. In einer zweiwöchigen Probephase wurden sowohl aktive als auch passive Orthesen intensiv erprobt. Da die aktive Variante (funktionelle Elektrostimulation (FES)-Schiene) zu erhöhter Auslösung der Spastik der Plantarflexoren führte, wurde eine passive Orthese (Dynamic Walk, Single Side Lateral, Fillauer Companies, Inc., Chattanooga, TN, USA) im weiteren Therapieverlauf verwendet (Abb. 2).

Nach der Testphase kam der Patient erneut ins Ganglabor zur Vergleichsmessung mit getragener Sprunggelenksorthese. Zu allererst ist die deutliche Erhöhung der Ganggeschwindigkeit aufgefallen, hier konnte nahezu eine Verdopplung gegenüber der Messung ohne Orthese gemessen werden (Tab. 2). Ebenso zeigten die Kadenz und die Schrittlänge links deutliche Verbesserungen. Vom vormals markanten Beistellschritt auf dieselbe Höhe, kam der Patient nun bis circa eine halbe Fußlänge vor den rechten Fuß, dabei setzte der linke Fuß im Initialkontakt mit der Ferse auf. Bezugnehmend auf die Hyperextension des Kniegelenks ist festzustellen, dass die Dauer der Hyperextension reduziert werden konnte, bei allerdings nach wie vor vorhandener maximaler Hyperextension von über 10°. Hier wurde für den weiteren Verlauf eine medikamentöse Spastikbehandlung diskutiert. Aus den sagittalen Bewegungskurven des Sprunggelenks geht hervor, dass der Patient mit Orthese den Fuß in der Schwungphase nicht mehr in Plantarflexionsstellung führt, sondern in Dorsalextension (Abb. 1). Das Zehenschleifen am Boden ist aufgehoben. Zudem konnte eine Verbesserung hinsichtlich der Supinationsstellung des linken Sprunggelenks in der ipsilateralen Schwungphase festgestellt werden, dadurch wird das Risiko des Hängenbleibens am rechten Standbein reduziert. In Folge berichtete der Patient, er müsse sich beim Gehen weniger auf die Bodenfreiheit und die Sturzgefahr konzentrieren und könne so Aufmerksamkeit, die zum Gehen benötigt wird, reduzieren.

**Table 2:** 

	1. Messung		2. Messung		
	Links	Rechts	Links	Rechts	Norm
Kadenz (Schritte/min)	28 ± 5	28 ± 6	43 ± 3	43 ± 3	116 ± 8
Ganggeschwindigkeit (m/s)	0,14 ± 0,03	0,15 ± 0,03	0,26 ± 0,03	0,26 ± 0,03	1,36 ± 0,17
Schrittlänge (m)	0,10 ± 0,04	0,50 ± 0,02	0,21 ± 0,03	0,53 ± 0,03	0,70 ± 0,06
Spurbreite (m)	0,14 ± 0,02	0,15 ± 0,01	0,15 ± 0,01	0,14 ± 0,01	0,08 ± 0,03
Monopedale Standphase (s)	0,53 ± 0,05	0,97 ± 0,04	0,43 ± 0,02	0,71 ± 0,07	0,43 ± 0,03
Bipedale Standphase (s)	3,06 ± 0,95	3,18 ± 1,02	1,68 ± 0,23	1,68 ± 0,22	0,20 ± 0,04
Ende der Standphase (%)	80 ± 5	87 ± 3	74 ± 4	84 ± 1	60 ± 2

Der Patient gab ein deutlich erleichtertes Ganggefühl beim Tragen der Dynamic-Walk-Orthese an. Die Schwungbeinphase ist dadurch besser, ein Zugewinn der Schrittlänge links konnte erreicht, das Nachschleifen des Fußes minimiert und das Einsetzen der Spastik verzögert werden. Zu Behandlungsbeginn trat bereits nach 5 min Gehen eine limitierende Spastik auf, gegen Ende der querschnittsspezifischen Rehabilitation und Anpassung der Hilfsmittel trat diese erst nach 15 min auf. In diesem Fallbeispiel konnte aufgrund der Ergebnisse der Bewegungsanalyse nicht nur das Gangbild verbessert, sondern auch eine Besserung des psychischen Wohlbefindens erreicht werden.

### Fallbeispiel 2

Im Rahmen einer stationären querschnittspezifischen Rehabilitation wurde ein 42-jähriger Patient mit der Diagnose einer inkompletten Tetraplegie sub C5 AIS D zur Bewegungsanalyse vorgestellt. Zum Zeitpunkt der Messung bestand die traumatisch bedingte inkomplette Lähmung seit 4 Jahren und 3 Monaten. Es sollte eine allgemeine Beurteilung des Gangbildes zur Unterstützung der weiteren Therapieplanung erfolgen. Der Patient beklagte eine generelle Verschlechterung des Gangbilds in den letzten 2 Jahren. Zusätzlich habe er während des Gehens Schmerzen im rechten Kniegelenk.

Im Muskelfunktionsbefund nach Janda [27] zeigte das linke Bein bessere Kraftwerte als die Gegenseite (Tab. 3). Die Tiefensensibilität sowie die passive Beweglichkeit der Gelenke der unteren Extremität waren unauffällig. Im Rahmen der Funktionsuntersuchung konnten keine Spastiken provoziert werden. Der Patient war zum Zeitpunkt der Messung noch nicht mit gangunterstützenden Hilfsmitteln versorgt.

**Table 3:** 

	Links	Rechts
M. iliopsoas	4	3
M. gluteus maximus	3	2
M. gluteus medius	4	4
M. quadriceps femoris	5	3
Mm. ischiocrurales	5	3
M. gastrocnemius	4	2
M. tibialis anterior	5	5
M. peroneus	5	5

Das Hauptproblem wurde der fehlenden Kontrolle des rechten Knie- und Sprunggelenks in der Sagittalebene während der Standphase zugeschrieben. Hier wird die Hauptursache in der deutlich verringerten Kraft der Plantarflexoren rechts mit 2/5 nach Janda gesehen.

**Figure 3:**
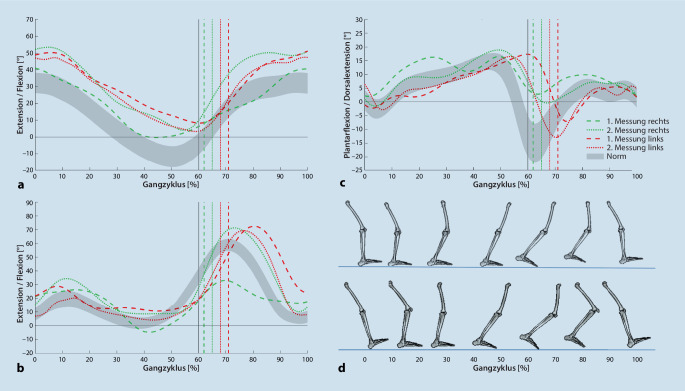

In der Standphase (Abb. 3 und 4) war der Patient nicht in der Lage, einer verfrühten Progression der Tibia entgegenzuwirken. Unter Belastung wurde das Sprunggelenk durch die muskuläre Insuffizienz zwischen 12–30 % des Gangzyklus (mittlere Standphase) in eine übertriebene Dorsalextension gedrückt, die Knieflexion wurde daher, ebenso wie die externen Knieflexionsmomente, verlängert beibehalten. Dies erfordert eine kontinuierliche Aktivität des M. quadriceps femoris [25], was zu dem vom Patienten angegebenen Knieschmerz führen könnte. Im weiteren Verlauf (30–40 % des Gangzyklus) wurde durch eine maximale Dehnung der Plantarflexoren eine weitere Progression der Tibia verhindert, wodurch das Kniegelenk in Extension bewegt wurde. Durch den zweigelenkigen M. gastrocnemius, der durch die Knieextension vermehrt unter Spannung gerät und die Dorsalextension des Sprunggelenks in physiologischer Weise verringert [27], kam es zu einer rückwärtsgerichteten Bewegung der Tibia. Aufgrund der geringen Kraft des M. gastrocnemius und seiner somit verminderten knieflektorischen Aktivität konnte eine Hyperextension des Kniegelenks nicht verhindert werden [6]. Dies führt zu einer erhöhten Belastung der dorsalen Bandstrukturen des Kniegelenks was langfristig zu Schmerzen führen kann. In der terminalen Standphase (bis 50 % des Gangzyklus) blieb die rechte Ferse aufgrund der geringen Kraft der Plantarflexoren bis zum Fußaufsatz des kontralateralen Beines auf dem Boden [25]. Der für diese Phase typische Spannungsaufbau der Achillessehne fand nicht statt. Dieser Spannungsaufbau leistet einen wichtigen Beitrag, die Tibia in der darauffolgenden Vorschwungphase (50 % des Gangzyklus bis „toe-off“) nach vorne zu transportieren und somit die Knieflexion einzuleiten [25].

**Figure 4:**
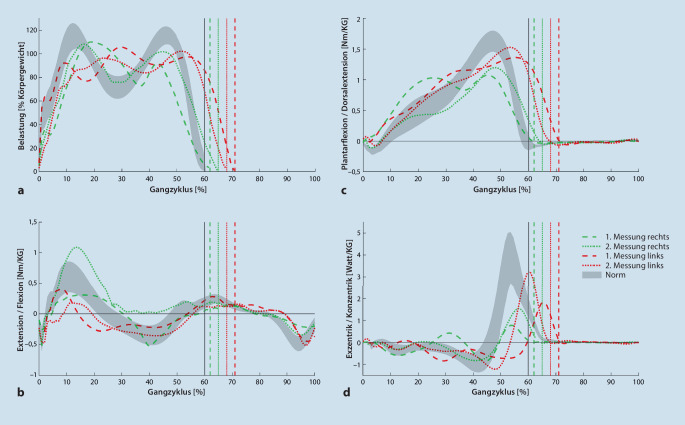

Die Knieflexion war in der Schwungphase deutlich reduziert, die Hüftflexion wurde verzögert eingeleitet (Abb. 3). In den Bodenreaktionskräften der kontralateralen linken Seite wurde durch einen zusätzlichen Kraftgipfel bei 30 % des linken Gangzyklus deutlich, dass die Geschwindigkeit des rechten Schwungbeins nicht ausreicht, um eine Entlastung des linken Standbeins zu ermöglichen (Abb. 4; [14]). Der im Anschluss stattfindende initiale Bodenkontakt wurde mit einer übertriebenen Knieflexion vorbereitet. Vermutlich diente diese Ausweichbewegung als Ausgleich für eine verminderte Koordination der abgeschwächten ischiokruralen Muskulatur, die in den ersten 10 % des Gangzyklus das Knie in 5° Flexion stabilisieren sollte, um eine Hyperextension ab dem initialen Bodenkontakt zu verhindern [11].

**Figure 5:**
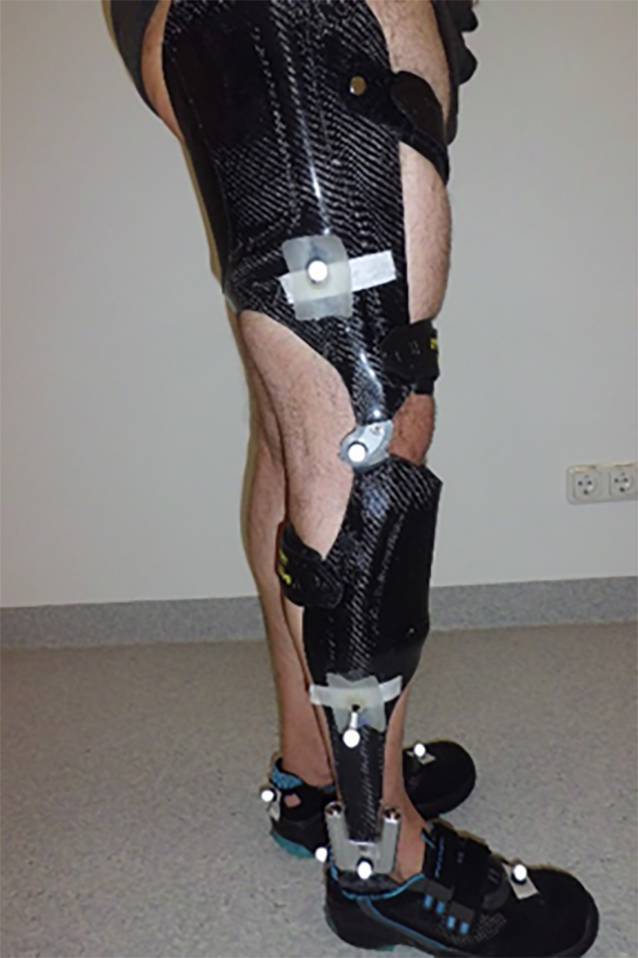

Aufgrund der Ergebnisse der Bewegungsanalyse erfolgte eine Versorgung mit einer maßgefertigten Orthese aus Faserverbundwerkstoff mit ventraler Unterschenkel- und dorsaler Oberschenkelanlage sowie Gelenken der Firma Fior & Gentz (Lüneburg, Deutschland) (Knie: Neuro Vario, Sprunggelenk: Neuro Swing) (Abb. 5). Die Evaluierung der Orthesenversorgung erfolgte 3 Monate nach der ersten Messung.

Durch die Orthese kam es zu einer Verbesserung der Symmetrie, sowohl in den Weg-Zeit-Parametern (Tab. 4) als auch in der sagittalen Gelenkkinematik und -kinetik (Abb. 3 und 4). Im initialen Bodenkontakt wurde die zuvor beobachtete übertriebene Flexion des rechten Kniegelenks um 5° verbessert. Dadurch konnte bei nahezu identischer Position des oberen Sprunggelenks in der Belastungsübernahme (bis 12 % des Gangzyklus) ein Abrollen über die Ferse stattfinden („heel rocker“). Die stoßdämpfende Knieflexion sowie die externen Flexionsmomente stellen sich zwischen 10–20 % des Gangzyklus als zu groß dar. Durch das rückverlagerte Neuro-Vario-Kniegelenk (Abb. 5) soll einer übermäßigen Flexion in dieser Phase entgegengewirkt werden, hierzu ist allerdings eine gute Vorpositionierung des Kniegelenks im initialen Bodenkontakt notwendig. In interdisziplinärer Absprache wurde hierfür ein physiotherapeutisches Gangtraining beschlossen. Zudem wurde von Seiten der Orthopädietechniker geplant, den Dorsalextensionsanschlag der Orthese härter einzustellen, um die übermäßige Knieflexion zwischen 10 und 20 % des Gangzyklus zu unterbinden. In der terminalen Standphase konnte die Hyperextension des Kniegelenks verhindert werden. Durch die Limitierung der Dorsalextension des Orthesengelenkes sowie die ventrale Schienenanlage am Unterschenkel konnte durch die entstehende Hebelwirkung ein Fersenhub im terminalen Stand erzeugt werden. Die daraus resultierende Verschiebung des Körperschwerpunkts nach anterior ermöglichte eine verbesserte Einleitung der Schwungphase in Knie- und Hüftgelenk [33]. Das rechte Bein konnte dynamischer nach vorne geschwungen werden, hiermit normalisierten sich im kontralateralen Standbein die Bodenreaktionskräfte.

**Table 4:** 

	1. Messung		2. Messung		
	Links	Rechts	Links	Rechts	Norm
Kadenz (Schritte/min)	91 ± 2	93 ± 3	94 ± 3	94 ± 3	116 ± 8
Ganggeschwindigkeit (m/s)	0,73 ± 0,03	0,74 ± 0,03	1,02 ± 0,03	1,01 ± 0,05	1,36 ± 0,17
Schrittlänge (m)	0,50 ± 0,01	0,46 ± 0,03	0,63 ± 0,02	0,67 ± 0,02	0,70 ± 0,06
Spurbreite (m)	0,13 ± 0,02	0,14 ± 0,02	0,11 ± 0,06	0,09 ± 0,04	0,08 ± 0,03
Monopedale Standphase (s)	0,53 ± 0,01	0,38 ± 0,02	0,48 ± 0,01	0,43 ± 0,02	0,43 ± 0,03
Bipedale Standphase (s)	0,42 ± 0,02	0,42 ± 0,01	0,40 ± 0,02	0,40 ± 0,02	0,20 ± 0,04
Ende der Standphase (%)	71 ± 1	62 ± 1	68 ± 1	65 ± 1	60 ± 2

## Fazit für die Praxis

- Objektive Messverfahren zur qualitativen Untersuchung des Gangbilds von inkomplett Querschnittverletzten werden bisher wenig eingesetzt.
- Veränderungen des Gangbilds nach einer Intervention können mittels markerbasierter Bewegungsanalyse objektiv nachgewiesen werden.
- Markerbasierte Bewegungsanalysen können Therapieentscheidungen positiv unterstützen.
- Die Messergebnisse sollten interdisziplinär besprochen werden.
- Eine umfassende Funktionsuntersuchung ist für die Interpretation der Bewegungsdaten zwingend erforderlich.

## Abkürzungen

AIS: American Spinal Injury Association Impairment Scale
BG: Berufsgenossenschaftlich
WISCI: Walking Index for Spinal Cord Injury
